# Breakpoint Mapping of Symptomatic Balanced Translocations Links the *EPHA6*, *KLF13* and *UBR3* Genes to Novel Disease Phenotype

**DOI:** 10.3390/jcm9051245

**Published:** 2020-04-25

**Authors:** Victor Murcia Pienkowski, Marzena Kucharczyk, Małgorzata Rydzanicz, Barbara Poszewiecka, Katarzyna Pachota, Marlena Młynek, Piotr Stawiński, Agnieszka Pollak, Joanna Kosińska, Katarzyna Wojciechowska, Monika Lejman, Agata Cieślikowska, Dorota Wicher, Agnieszka Stembalska, Karolina Matuszewska, Anna Materna-Kiryluk, Anna Gambin, Krystyna Chrzanowska, Małgorzata Krajewska-Walasek, Rafał Płoski

**Affiliations:** 1 Department of Medical Genetics, Medical University of Warsaw, 02-106 Warsaw, Poland; victorabel.murcia@gmail.com (V.M.P.); mrydzanicz@wum.edu.pl (M.R.); stawinski84@gmail.com (P.S.); poli25@wp.pl (A.P.); samocko@wp.pl (J.K.); 2 Department of Medical Genetics, The Children’s Memorial Health Institute, 04-730 Warsaw, Poland; M.Kucharczyk@ipczd.pl (M.K.); K.Pachota@ipczd.pl (K.P.); mlynek.marlena@gmail.com (M.M.); A.Cieslikowska@ipczd.pl (A.C.); D.Wicher@ipczd.pl (D.W.); K.Chrzanowska@ipczd.pl (K.C.); m.krajewska-walasek@ipczd.pl (M.K.-W.); 3 Institute of Informatics, Faculty of Mathematics, Informatics and Mechanics, University of Warsaw, 02-097 Warsaw, Poland; b.poszewiecka@gmail.com (B.P.); aniag@mimuw.edu.pl (A.G.); 4 Department of Pediatric Hematology Oncology and Transplantology, University Children’s Hospital, 20-093 Lublin, Poland; kate.woj@hotmail.com; 5 Department of Pediatric Hematology Oncology and Transplantology, Medical University of Lublin, 20-093 Lublin, Poland; lejmanm@poczta.onet.pl; 6 Department of Genetics, Wroclaw Medical University, 50-368 Wroclaw, Poland; stembalska8@gmail.com; 7 Department of Medical Genetics, University of Medical Sciences, 60-806 Poznan, Poland; k.matuszewska@genesis.pl (K.M.); akiryluk@ump.edu.pl (A.M.-K.); 8 Centers for Medical Genetics GENESIS, Grudzieniec, 60-406 Poznan, Poland

**Keywords:** *EPHA6*, *KLF13*, *UBR3*, de novo balanced aberrations, mate-pair sequencing, developmental delay

## Abstract

De novo balanced chromosomal aberrations (BCAs), such as reciprocal translocations and inversions, are genomic aberrations that, in approximately 25% of cases, affect the human phenotype. Delineation of the exact structure of BCAs may provide a precise diagnosis and/or point to new disease loci. We report on six patients with de novo balanced chromosomal translocations (BCTs) and one patient with a de novo inversion, in whom we mapped breakpoints to a resolution of 1 bp, using shallow whole-genome mate pair sequencing. In all seven cases, a disruption of at least one gene was found. In two patients, the phenotypic impact of the disrupted genes is well known (*NFIA, ATP7A*). In five patients, the aberration damaged genes: *PARD3, EPHA6, KLF13, STK24, UBR3, MLLT10* and *TLE3*, whose influence on the human phenotype is poorly understood. In particular, our results suggest novel candidate genes for retinal degeneration with anophthalmia (*EPHA6*), developmental delay with speech impairment (*KLF13*), and developmental delay with brain dysembryoplastic neuroepithelial tumor (*UBR3*). In conclusion, identification of the exact structure of symptomatic BCTs using next generation sequencing is a viable method for both diagnosis and finding novel disease candidate genes in humans.

## 1. Introduction

Next generation sequencing (NGS) marked a new era in identification of rare diseases in humans [1]. However, whereas the most straightforward approach consists of analyzing single nucleotide variants (SNVs) and small insertions and deletions, the detection of structural variants (SVs) is less easy [2]. This poses a particular problem for the detection of balanced chromosomal translocations (BCTs), 26.8% of which are associated with pathogenic phenotype [3], making these patients an interesting group for the identification of new genes/loci linked to human disease.

BCTs and balanced inversions may lead to disease via three main mechanisms: (1) direct breaking of a gene which leads to haploinsufficiency [4], (2) creation of fusion genes, which may cause the expression of a new chimeric gene or affect regulatory elements—fusion genes have mostly been associated with cancer; however they are also implicated in developmental delay [5,6]; (3) change in expression of genes that were not directly damaged by the SVs, but were affected due to their location in the disrupted topologically associating domain (TAD, position effect) [7].

Here, we present six cases of de novo BCTs and one case of de novo balanced inversion in symptomatic patients. In each case, we were able to identify the exact structure of the breakpoint using shallow whole genome mate-pair sequencing. We propose that the analyzed balanced chromosomal aberrations (BCA) may be responsible for the disease in the patients.

Below, we present the clinical description of all the analyzed patients. Written consent for the research procedure was obtained from the legal guardians of the patients. In the probands accepted for the study, de novo BCTs or balanced inversions were identified through karyotyping of the patients and the parents. Other chromosomal aberrations in the patients were excluded using comparative genomic hybridization array (aCGH).

### 1.1. Proband 1

The boy was born at 39 weeks of the first pregnancy with an Apgar score of 10, weight 3130 g (25 to 50 c), height 53 cm (>90 c) and head circumference 35 cm (75 c). At birth, defects of the eyeballs were identified: right anophtalmia, retinal degeneration and persistent pupillary membrane in the left eye. Additionally, developmental delay (DD) was observed. The boy started talking and sitting at 12 months and started walking at the age of 3 years. The boy was referred for genetic counselling at 4 months, because of the birth defects of the eyeballs. At the age of 7 years, he spoke only short sentences. He attends a public school for blind children and presents specific abnormal behavior (shaking head and hands). On MRI, apart from right anophtalmia, elongated left eyeball and very thin optic nerves were observed.

### 1.2. Proband 2 

The boy was born at 42 weeks of the first pregnancy with an Apgar score of 10, weight 3000 g (25 to 50 c), height 53 cm (>90 c) and a head circumference 35 cm (75 c). The patient was referred for genetic counselling due to hypotonia and dysmorphic features at the age of 2 years. Dysmorphic features included: short stature, low body weight, deep philtrum, frontal bossing, high frontal hairline, epicanthus, asymmetric palpebral fissures, down slanting palpebral fissures, flat, full cheeks, small chin.

### 1.3. Proband 3

The boy was referred at the age of 3.5 years for genetic counselling because of DD. At the time, he did not yet speak. He was born from the second pregnancy of young, healthy and non-consanguineous parents. His brother from the first pregnancy was intellectually normal but was affected by ventricular septal defect (VSD), atrial septal defect (ASD II) and submucosal cleft palate. The proband was born at 39th week of pregnancy with an Apgar score of 10, weight 3300 g (50 c), height 53 cm (>90 c) and a head circumference of 35 cm (50 c). His motoric development was nearly normal—he began to sit at 7 months and to walk at 14 months. During the last examination, at the age of 7.5 years, coarse facial features, short palpebral fissures, nasal bridge, cup-like ears, thin upper lip, palate high set, prognathism and narrow nails were noted. Cutis marmorata, especially on the trunk, was observed. Overall moderate DD was diagnosed. MRI revealed a constellation of abnormalities, such as left periventricular nodular heterotopias, septum pellucidum cyst and thinning of corpus callosum.

### 1.4. Proband 4

A 17 years old, a girl was referred for clinical evaluation to the Centre for Medical Genetics, GENESIS, in Poznan, Poland. The proband was from an uneventful pregnancy with natural delivery at 37 weeks, Apgar 9, birth weight 3200 g. She was the second child born to healthy non-consanguineous parents. There was a maternal history of miscarriages, with the first pregnancy miscarried at 16 weeks, and there were three premature deliveries from pregnancies II, III and IV at weeks 20, 23 and 23, respectively. The proband has one healthy sister from the fifth pregnancy. The family history included a first-degree cousin with intellectual disability and a congenital heart defect.

In the first two days of life, abnormal respiratory function was observed. At age 20 months, epileptic seizures occurred. Delayed speech development was also observed. Magnetic resonance imaging (MRI) of the brain at the age of 3.5 was normal. At age 13, she was hospitalized again and a diagnosis of brain dysembryoplastic neuroepithelial tumor was established. The girl was operated on, and after the surgery the seizures resolved. Psychological examination revealed severe intellectual disability. We also observed delayed speech and repetitive hand movements. At the age of 17, craniofacial dysmorphic features included only slight facial asymmetry and high-arched palate. *MECP2* gene was sequenced, but no pathogenic variants were detected.

### 1.5. Proband 5

A 9-month-old girl with DD and dysmorphic features was admitted for evaluation. She was the third child of non-consanguineous, healthy parents, born at 39 weeks of gestational age by complicated vaginal delivery (true umbilical cord knot, green amniotic fluid). Maternal hypothyroidism and decreased fetal movements during pregnancy were noticed. Apgar score was 9 and 10 at first and fifth minutes after birth, respectively. Birth weight was 3350 g, head circumference 35 cm. After three months of normal development, progressive hypotonia, low spontaneous activity and failure to thrive had occurred. Physical examination revealed a pale, redundant skin, prominent forehead and sparse, coarse hair. Neurological examination showed a hypoactive baby, without social grinning, who could not maintain visual contact nor follow objects. Pupils were normal and reactive to light. Deep tendon reflexes were brisk with significant hypotonia. Optic microscopy of a hair sample disclosed pili torti. Neuroimaging (magnetic resonance) findings showed delayed myelination, mild ventriculomegaly, thin corpus callosum and vascular tortuosity. An abdominal ultrasound revealed multiple bladder diverticula. Based on these findings, the diagnosis of Menkes disease was made and confirmed by low serum ceruloplasmin and copper concentration.

### 1.6. Proband 6

The patient is a female born through natural delivery from the first pregnancy at risk of miscarriage, at 39 weeks of gestation, with a weight of 3140 g and a body length of 53 cm and with an Apgar score of 10. When the patient was 18 months old, a delay in her speech development was observed following an episode of febrile convulsions. Neurological observation of the patient revealed generalized laxity. EEG examination showed no abnormalities apart from spindle deficits. MRI scan was normal. A psychological assessment of the child revealed no features of autism. The speech pathology assessment of the patient carried out during the first years of her life showed lack of speech, poor vocalization and no interest in verbal communication with the environment. From the age of three, the child has attended kindergarten, where she requires assistance from a support teacher. The child’s speech development is delayed and she still requires speech therapy. Her vocabulary is poor. At the age of six she was referred to a genetics department with suspected Sotos syndrome.

### 1.7. Proband 7

The girl was born from the second pregnancy in the 40th week of gestation with an Apgar score of 10, weight 2890 g (10–25 c), height 52 cm (90 c), head circumference 35 cm (50 c). The delivery was resolved by caesarean section due to breech position. From the beginning, generalized hypotonia and developmental delay were observed. She sat at 18 months and started to walk two months later. She never spoke any words. Until 9 years, her weight was below the third centile. Due to autism and outbursts of aggression, she was put on a strict diet without gluten, milk or sugar. At the age of 13 years, the girl was referred to the Genetic Counselling Unit of the Children’s Memorial Health Institute, because of autistic features and lack of speech. Overall, global DD with no dysmorphic features was diagnosed.

## 2. Materials and Methods

The methods used in this project have been described previously [8]. In short, DNA was extracted using the salting out method from peripheral blood of patients with de novo BCTs, previously identified through karyotyping. Absence of additional deletions/duplications was confirmed using aCGH in all patients and their parents. Shallow genomic sequencing was carried out with Mate Pair Library Preparation Kit (Illumina, San Diego, CA, USA) with insert size 2–4 kb. Next, sequencing was performed on Illumina HiSeq 1500 (2 × 100 bp). For every patient, approximately 30 million reads were mapped to the reference genome (hg19) using bowtie2, leading to an average genomewide coverage of 5×. The *locus* of the translocation was identified using a custom made “R” package detecting discordant reads (paired reads where reads map to different chromosomes). Only clusters with at least 15 similar discordant reads were considered for further verification. The exact structure of the BCTs was verified by Sanger sequencing or deep amplicon sequencing (Nextera XT DNA Preparation Kit (Illumina), sequenced on Illumina Hiseq 1500 (2 × 100 bp). For the region flanking every identified breakpoint topologically associating domains (TADs) were analyzed using data obtained from Hi-C experiments on human GM12878 B-lymphoblastoid cells. The visualization of TADs was conducted using the TADeus web service [9]. 

Ethical approval for the study was granted by the Ethical Committee at the Medical University of Warsaw (KB/127/2017) on 08.06.2017.

## 3. Results

**Figure 1:**
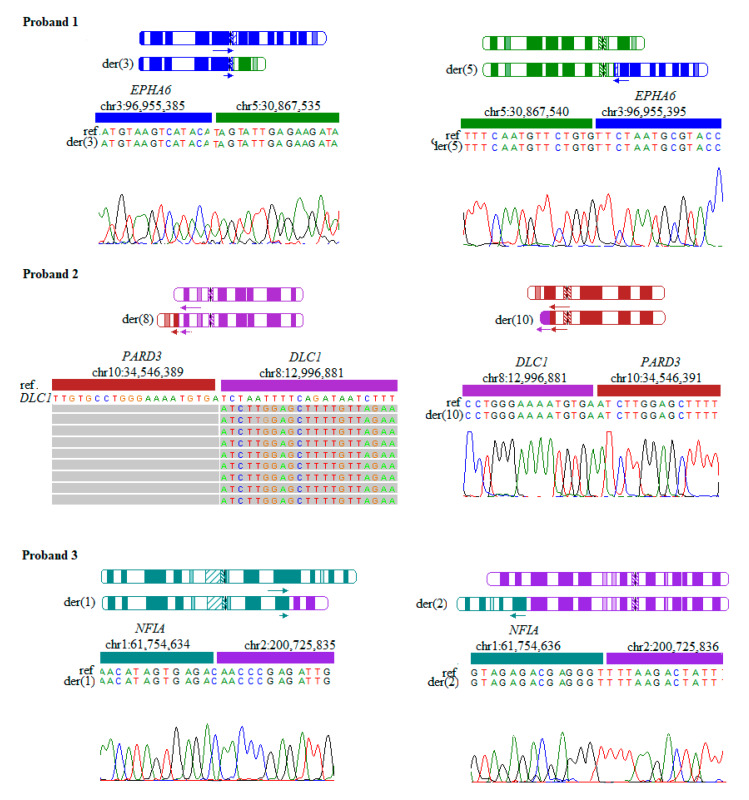

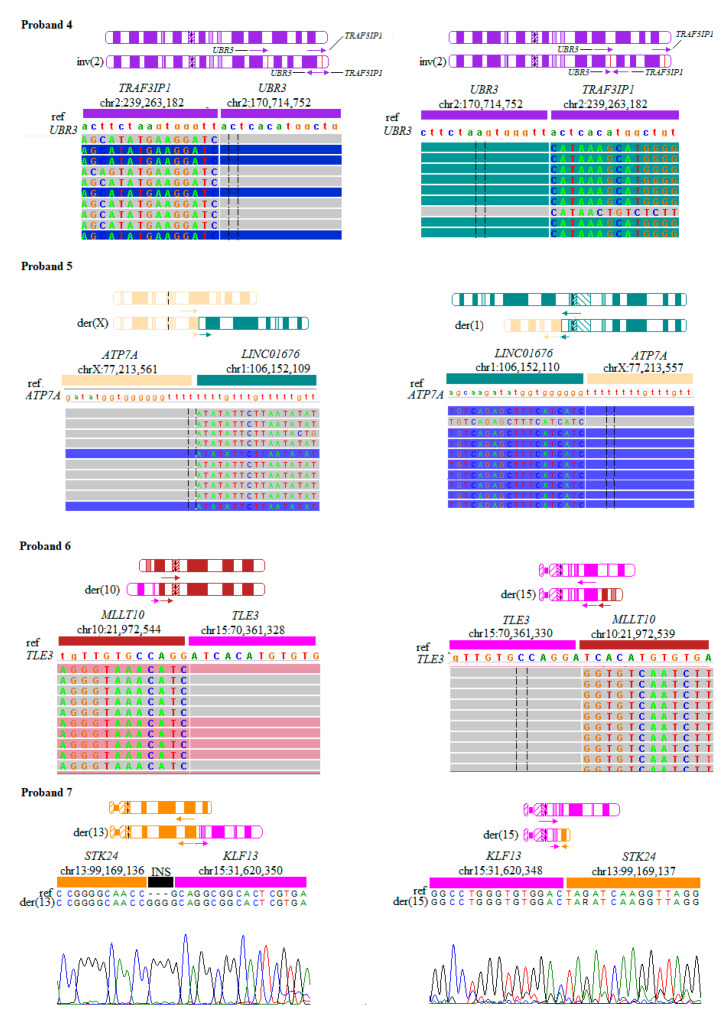
Results from Sanger sequencing and/or deep amplicon sequencing with NexteraXT library of the balanced chromosomal aberrations (BCAs) breakpoints. The figure for every patient includes: in the upper part, a schematically shown normal chromosome and below a chromosome containing the BCAs. Arrows indicate the orientation of the genes affected by translocation. The length of the arrows approximately corresponds to the length of the disrupted coding sequences. The lower part shows the sequencing results. For every BCA, both breakpoints are shown. In deep amplicon sequencing results, only split reads are shown (reads that present the exact structure of the breakpoint). One part of these reads fully maps to the reference genome, while the other part differs from the reference. In Proband 2 an inframe fusion gene might be created.

The exact structure of the BCAs of each patient including break-point positions and disrupted genes is shown in Table 1. In five out of seven patients (pat. 1,2,4,6,7) the chromosomal aberration disrupted at least one gene without a defined impact on human phenotype in OMIM [10] (*EPHA6, PARD3, UBR3, MLLT10, TLE3, STK24, KLF13*). In addition, in two patients (patients 3,5) the translocations disrupted *NFIA* and ATP7A, which are a known cause of developmental delay and Menkes disease, respectively. The structure of TADs that were directly disrupted by the translocations are shown for every proband in File S1. The exact structure of the BCAs verified by Sanger sequencing or deep amplicon sequencing are shown in Figure 1.

## 4. Discussion

In seven analyzed patients with de novo balanced chromosomal rearrangements (six BCTs, and one inversion) we identified 11 directly disrupted genes: *EPHA6, PARD3, DLC1, NFIA, UBR3, TRAF3IP1, ATP7A, MLLT10, TLE3, STK24, KLF13.* In two patients, the function of the disrupted genes is well established (*NFIA, ATP7A*). In the remaining five patients, the de novo event disrupted genes with a poorly defined function (*EPHA6, PARD3, DLC1, UBR3, MLLT10, TLE3, STK24, KLF13*). DOMINO scores, LOEUF scores, the number of loss of function variants in gnomAD and the expected number of loss of function variants in gnomAD for all the genes are presented in Table S1 [11]. The most interesting genes that were damaged by the BCTs are: *EPHA6* (the first case with an optic phenotype in humans), *UBR3* (the second case with developmental delay in humans), *KLF13* (second case with developmental delay in humans). In the patient with a BCT disrupting *MLLT10* and *TLE3,* it is unclear which gene is responsible for the phenotype, nevertheless both genes have few possibly pathogenic variants reported in humans (all linked to autism spectrum disorder) and none of them are similar to the proband phenotype. In silico analysis of the TADs did not identify any clear candidates that could be responsible for the phenotype of the patients through position effect (File S1).

In proband 1, a boy with DD and anophthalmia, the BCT disrupted *EPHA6* gene. *EPHA6* is an ephirin receptor belonging to a subfamily of tyrosine kinases receptors. *EPHA6* LOEUF = 0.357 and DOMINO = 0.67 are consistent with autosomal dominant inheritance pattern of a disease caused by a loss-of-function variant. *EPHA6* has been linked to various processes including retinal axon guidance, retinal ganglion cells (RGCs) organization during brain development [12,13] and neural development [14]. Knockout of *EphA6*^−^ in mice causes learning and memory impairment [15]. In mice, *EphA6* is expressed in the posterior segment of the eye, including the retina [16].

In humans, *EPHA6* impact on the phenotype remains unclear, nevertheless, according to the Human Protein Atlas [17] *EPHA6* is highly expressed in the brain, consistent with the retinal axon guidance function of the gene product [12]. The phenotype of our patient suggests that the retinal degeneration and anophthalmia may be related to the defect of *EPHA6* through disruption of RGC organization and retinal axon guidance. Mild DD present in our patient is also compatible with the phenotype of *EphA6*^-/-^ mice (learning and memory impairment). Interestingly, database STRING predicted interactions between *EPHA6* and *EFNA5* (MIM: 601535)*,* a gene previously linked by our team to an ocular phenotype (bilateral cloudy cornea) [8,18].

In proband 2, a boy with hypotonia and dysmorphic features, the translocation disrupted two genes *DLC1* and *PARD3*. It is possible that a fusion gene was created as both genes are transcribed from the reverse strand and the translocation affects the *p* arms of both chromosomes (i.e., the orientation of the fused genes allows for a chimeric gene to be transcribed). Furthermore, the putative fusion of the transcripts is predicted to be in-frame. *DLC1* encodes a GTPase-activating protein for the small GTPases, that stops their signaling [19]. In humans, *DLC1* defects, depending on the variant, have been associated with autosomal dominant congenital heart disease or nephrotic syndrome [20,21]. Curiously, the patient’s phenotype does not match any of these. Since the number of reported missense variants in *DLC1* is relatively small (*n* = 26), it is possible that the disease spectrum of this gene is not yet fully known [20,21]. Furthermore, all but four previously reported cases were associated with missense variants, whereas the BCT in our proband is predicted to cause loss of function. Two out of four previously reported *DLC1* nonsense variants were associated with neural tube defect [22]. Notably, *DLC1* has a high DOMINO score = 0.7 which supports that monoallelic loss of function variants of this gene may be pathogenic. Another possibility explaining the lack of the *DLC1* phenotype is that while the translocation disrupts the longest transcript (NM_182643), two shorter transcripts encoding potentially functional proteins remain unaffected. Nevertheless, we cannot discard the possibility that the translocation disrupts *in cis* regulatory sequences of the shorter isoforms.

*PARD3* encodes a protein from the PARD family, which is involved in asymmetric cell division and polarized cell growth [23]. *Pard3* has been linked to dendritic spine development in rat hippocampal neurons [24]. Regarding the impact on human phenotype, there are five publications describing six variants linking *PARD3* to autism spectrum disorder (ASD) [25,26,27,28,29]. However, only one variant has been reported as “high confidence” by HGMD. Furthermore, two *PARD3* variants were implicated in neural tube defects by a case control association study [30]. Thus, at present it is difficult to explain the phenotype of our patient by the available information on effects of *DLC1* and *PARD3* defects.

Proband 3, a boy with DD, had one gene damaged by the translocation: *NFIA*. *NFIA* defects are a well-established cause of brain malformation with or without urinary tract defect [31,32]. Phenotype of our patient is fully consistent with these data.

In Proband 4, a girl with severe intellectual disability and a cured brain dysembryoplastic neuroepithelial tumor, we identified an inversion that disrupted two genes: *UBR3* and *TRAF3IP1*. The inversion did not create a fusion gene as both genes are transcribed from the forward strand (the orientation of the fused genes is incorrect for a chimeric gene to be created). *TRAF3IP1* defects cause autosomal recessive Senior–Loken syndrome [33]. Since the inversion disrupts a single copy of *TRAF3IP1,* it is unlikely that phenotype is caused by the defect of this gene. On the other hand, *UBR3* (LOEUF = 0.357, DOMINO = 0.7) is an interesting candidate as it encodes an ubiquitin ligase whose defects, based on six described variants, have been linked to ASD or congenital heart disease [26,28,34,35]. Additionally, one male patient with a heterozygous stop codon in *UBR3* and developmental delay (phenotype not precisely described) has been reported [35]. *UBR3* is a positive regulator of Hedgehog signaling pathway, which is also associated with DD [36], making it a plausible candidate gene in our patient. *UBR3* is expressed in the sensory epithelium or neurons of all major sensory systems, including olfaction. STRING pointed to one gene *FBXO11* (MIM: 312180), which probably interacts with *UBR3*, and is recognized as causing autosomal dominant intellectual developmental disorder with dysmorphic facies and behavioral abnormalities (MIM: 618089), making *UBR3* an even more probable cause of the patient phenotype [16,37]. UBR3 polyubiquitylates multiple targets, including APE1, an essential human protein involved in DNA damage repair and regulation of transcription. Because UBR3 regulates cellular levels of APE1 required for genome stability [38], the disruption of *UBR3* may have also contributed to the occurrence of neuroepithelial malignancy in our patient.

In Proband 5, a girl with Menkes disease, the BCT disrupted the *ATP7A* gene. *ATP7A* variants cause X-linked recessive (XLR) Menkes disease, occipital horn syndrome and spinal muscular atrophy [39,40,41,42]. Typically, XLR diseases are present only in male patients, while the our proband is female. However, there have been five reports of Menkes disease affecting female patients with translocations that damaged *ATP7A* [43]. The disease in these cases is probably caused by inactivation of the X chromosome skewed towards leaving the derived chromosome X functional (the opposite scenario leads to partial monosomy of the autosome involved in the BCTs which induces cell death [44]). We conclude that it is highly probable that the identified translocation is responsible for the phenotype of the patient.

In Proband 6, a girl with speech delay, two genes have been disrupted *MLLT10* and *TLE3*. A fusion gene that may have been created as *MLLT10* is transcribed from the forward strand, while *TLE3* from the reverse strand and the translocation disrupts the *p* arm on chromosome 10 and the q arm on chromosome 15. However, the breakpoint leads to a frameshift mutation caused by the disruption of the last codon in the *MLLT10* gene between exons 15 and 16. The disease in the patient may be due to the disruption of either of the genes as both have high DOMINO and low LOEUF (*MLLT10 -* DOMINO = 0.72, LOEUF = 0.182; *TLE3 -* DOMINO = 0.99, LOEUF = 0.073). *TLE3* is a transcriptional co-repressor from transducin-like enhancer family of proteins. *TLE3* knockout mice died in utero, due to improper cell differentiation of trophoblast giant cells. In humans, one de novo *TLE3* variant has been reported as potentially causing autism [26]. The *MLLT10* gene is responsible for maintaining epigenetic balance through histone methylation (H3K79me). In mice, *Mllt10* functions in correct midfacial formation [45]. In humans, two variants of *MLLT10* have been linked to ASD [28,46]. Such data make it difficult to point to the gene(s) responsible for the phenotype of the patient. However, based on the number of loss of function variants, it is possible that *TLE3* has a bigger impact than *MLLT10* (Table S1). Additionally, according to the STRING database, *TLE3* inhibits activation of transcription factors in Wnt signaling pathway through CTNNB1 [16]. Interestingly, *CTNNB1* (MIM: 116806) has been linked to autosomal dominant neurodevelopmental disorder with spastic diplegia and visual defects (MIM: 615075), making *TLE3* an even more plausible candidate for impacting the patient phenotype [47].

Proband 7, a girl with global DD had a translocation that damages two genes: *STK24* and *KLF13*. A fusion gene cannot be created, as both genes are located in the q arm of their respective chromosomes, and *STK24* is transcribed from the reverse strand, while *KLF13* is transcribed from the forward strand. Both genes are poorly understood in the context of human disease and their relative impact on our patient’s phenotype cannot be determined. *STK24* is highly expressed in the brain [17] and has been linked to neuronal migration [48], Whereas *STK24* DOMINO score (0.34) argues against its dominant effect, the relatively low LOEUF score (0.580) suggests that monoallelic loss of function of *STK24* could be pathogenic. *KLF13* has a high DOMINO score (0.96) and relatively low LOEUF (0.931), both consistent with a dominant effect*. KLF13* is expressed in the brain [49] and is one of the genes deleted in the 15q13.3 deletion syndrome. Furthermore, according to STRING, KLF13 interacts with SIN3A (MIM: 607776) and EP300 (MIM: 602700), which are both associated with syndromic DD [50,51].

## 5. Conclusions

Our results emphasize that the identification of the exact structure of BCTs is useful for diagnosis, as well as for finding new disease loci. While, in some cases, it may be difficult to annotate a phenotype to one disrupted gene, it is still informative to restrict the pathological phenotype of the patient to two genes.

## Figures and Tables

**Table 1:** Summary of the results of all the patients.

Proband	Karyotyping Results	Break-Point Positions	Phenotype	Disrupted Gene(s)
1	46,XY,t(3;5)(q11.2;p13.1)	chr3:96,955,385/chr5:30,867,535 chr3:96,955,395/chr5:30,867,540	Developmental delay, anophthalmia	*EPHA6* (MIM: 600066)
2	46,XY, t(8;10)(p22;p11.21)	chr10:34,546,389/chr8:12,996,881 chr8:12,996,881/chr10:34,546,391	Hypotonia, dysmorphic features	*PARD3* (MIM: 606745) *DLC1* (MIM: 604258)
3	46,XY,t(1;2)(p31.3;q33.1)	chr1:61,754,634/chr2:200,725,835 chr1:61,754,636/chr2:200,725,835	Developmental delay	*NFIA* (MIM: 600727)
4	46,XX,inv(2)(q31.1;q37.3)	chr2:170,714,752/chr2:239,263,182	Severe mental retardation, cured brain dysembryoplastic neuroepithelial tumor	*UBR3* (MIM: 613831) *TRAF3IP1* (MIM: 607380)
5	46,X,t(X;1)(q21.1;p21.1)	chrX:77,213,561/chr1:106,152,109 chrX:77,213,557/chr1:106,152,110	Menkes disease	*ATP7A* (MIM: 300011)
6	46,XX,t(10;15)(p12.31;q23)	chr10:21,972,544/chr15:70,361,328 chr10:21,972,539/chr15:70,361,330	Speech delay	*MLLT10* (MIM: 602409) *TLE3* (MIM: 600190)
7	46,XX,t(13;15)(q32.2;q13.3)	chr13:99,169,136/chr15:31,620,350 chr15:31,620,348/chr13:99,169,137	Developmental delay	*STK24* (MIM: 604984) *KLF13* (MIM: 605328)

## Supplementary Materials

The following are available online at https://www.mdpi.com/2077-0383/9/5/1245/s1, File S1 TADs structure and characteristics of genes present in the disrupted domain for each patient, including data from: ExAC ([52],pLI), inheritance from OMIM ([10], AD—Autosomal dominant inheritance, AR—Autosomal recessive inheritance) and mouse phenotype [16]. Table S1 Characteristics of genes disrupted by BCT in studied probands. If OMIM data was positive, further analysis of HGMD data was not conducted. Only genes linked to high confidence phenotypes are shown from HGMD. DOMINO P (AD)—A score derived from machine learning to predict genes associated with dominant disorders, the higher the score, the higher the probability that the gene causes a disease with autosomal dominant inheritance. LOEUF score—Lower values indicate low tolerance of loss of function variants in the gene [53]. AD—Autosomal dominant, AR—Autosomal recessive, LoF—Loss of function.
